# Pressure-imposed changes of benzoic acid crystals

**DOI:** 10.1007/s00894-015-2635-z

**Published:** 2015-03-13

**Authors:** Piotr Cysewski

**Affiliations:** Department of Physical Chemistry, Collegium Medicum of Bydgoszcz, Nicolaus Copernicus University in Toruń, Kurpińskiego 5, 85-950 Bydgoszcz, Poland

**Keywords:** Aromaticity, Benzoic acid, Crystal, *C*_2_^2^(8) synthon, High-pressure, HOMA, Lattice energy

## Abstract

Structural and energetic properties of benzoic acid crystals at pressure elevated from ambient condition up to 2.21 GPa were characterized. The directly observed variations of cell parameters and consequently cell volume are associated with many other changes including energetic, geometric, and electronic characteristics. First of all the non-monotonous change of lattice energy are noticed with the rise of pressure since the increase of stabilization up to 1GPa is followed by systematic decrease of lattice energies after extending the hydrostatic compression. There is also an observed increase of *C*_2_^2^(8) synthon stabilization interaction with increase of pressure. The lattice response rather than interaction within synthons are source of observed pressure-related trend of lattice energy changes. The energy decomposition analysis revealed that the total steric interactions determine the overall trend of lattice energy change with the rise of pressure. Besides geometric aromaticity index was used as a measure of geometric changes. Serious discrepancies were noticed between HOMA values computed with the use of experimental and optimized geometries of the ring. Even inclusion of uncertainties of experimental geometries related to limited precision of X-ray diffraction measurements does not cancel mentioned discrepancies. Although HOMA exhibit similar trends at modest pressures the diversity became surprisingly high at more extreme conditions. This might suggest limitations of periodic DFT computations at elevated pressures and the experimentally observed breaking of molecules at very high pressures will probably not be accounted properly in this approach. Also limitation of direct use of experimental geometries were highlighted.

**Electronic supplementary material:**

The online version of this article (doi:10.1007/s00894-015-2635-z) contains supplementary material, which is available to authorized users.

## Introduction

High-pressure crystallography became an important tool providing valuable information in material sciences [1, 2]. Although ambient conditions applied to our live environment are common experimental setups of crystallographic measurements, as can be directly inferred from CSD content [3], many interesting condensed-phase phenomena involve extreme conditions [4, 5]. Areas of interests of pressure-related structural changes are very broad and encompass among others the geoscience/astrophysics studies [6], syntheses of super-hard materials [7, 8], the search for new polymorphic forms of active pharmaceutical ingredients [9, 10], energetic materials characteristics [11], new chemical reactions [12, 13] and phase transitions [14]. It is worth emphasizing that properties of substances at extreme conditions are generally difficult to predict. High hydrostatic pressure was applied to various compounds [1, 2] including simple one element crystals, variety of organic solids, and even biologically-relevant systems [15]. Pressure is a very useful probe of the molecular structure as it drives materials to states of lower volume. Since the molecular crystals of organic compounds are stabilized mainly by weak intermolecular interactions they are rather easily compressible and consequently reduce the volume by several-fold factor. This in turn can lead to rearrangement of crystal constituents, generation of new polymorphs, alteration of molecular conformations, and imposing chemical reactions like polymerization [12, 13]. Among crystals studied at non-ambient pressure the structure of benzoic acid (BA) was characterized up to 2.25 GPa at 296 K [16]. Benzoic acid is important, because as the simplest aromatic carboxylic acid it can represent a model compound of many active pharmaceutical ingredients as for example salicylic acid, aspirin, diflunisal, flufenamic acid, and lasalocid. Furthermore, the crystal structure of BA is attractive since it comprises common *C*_2_^2^(8) synthon. BA crystallizes in the monoclinic system (space group P21/c) with the unit cell containing two pairs of planar pairs stabilized by *C*_2_^2^(8) synthon. Sets of such dimers form series of parallel planes [17, 18]. Several authors studied pressure-induced changes in H-bonded dimers [16, 19–21]. Interestingly the pressure can impose serious structural changes and among them twisting of molecular orientation, decreasing O-O distance and reordering of H atoms in the hydrogen bonds in *C*_2_^2^(8) synthon can occur above 0.30 GPa. Experiments performed on single crystals compressed up to 33 GPa confirmed that significant changes in the H-bonded dimer structure can occur including the breaking of bonds and formation of new chemical compounds. As a consequence of this, above 15 GPa inhomogeneous color changes of the crystal to yellow/brownish, growth of luminescence, and serious alterations of other spectroscopic properties are observed. At very high pressure the breaking of phenyl ring occurs, which is also typical for other aromatic rings such as benzene [22] and pyrene [23].

The aim of this paper is the quantitative analysis of intermolecular interactions alterations imposed by hydrostatic pressure on benzoic acid crystals. Since there are only seven records in CSD documenting in-situ high-pressure crystallization this work is restricted only to interval of pressures form 0.1 GPa up to 2.21 GPa. To the author’s best knowledge this is the first study of pressure related changes of benzoic acid crystal energies.

## Method

### Crystals optimization

**Fig. 1:**
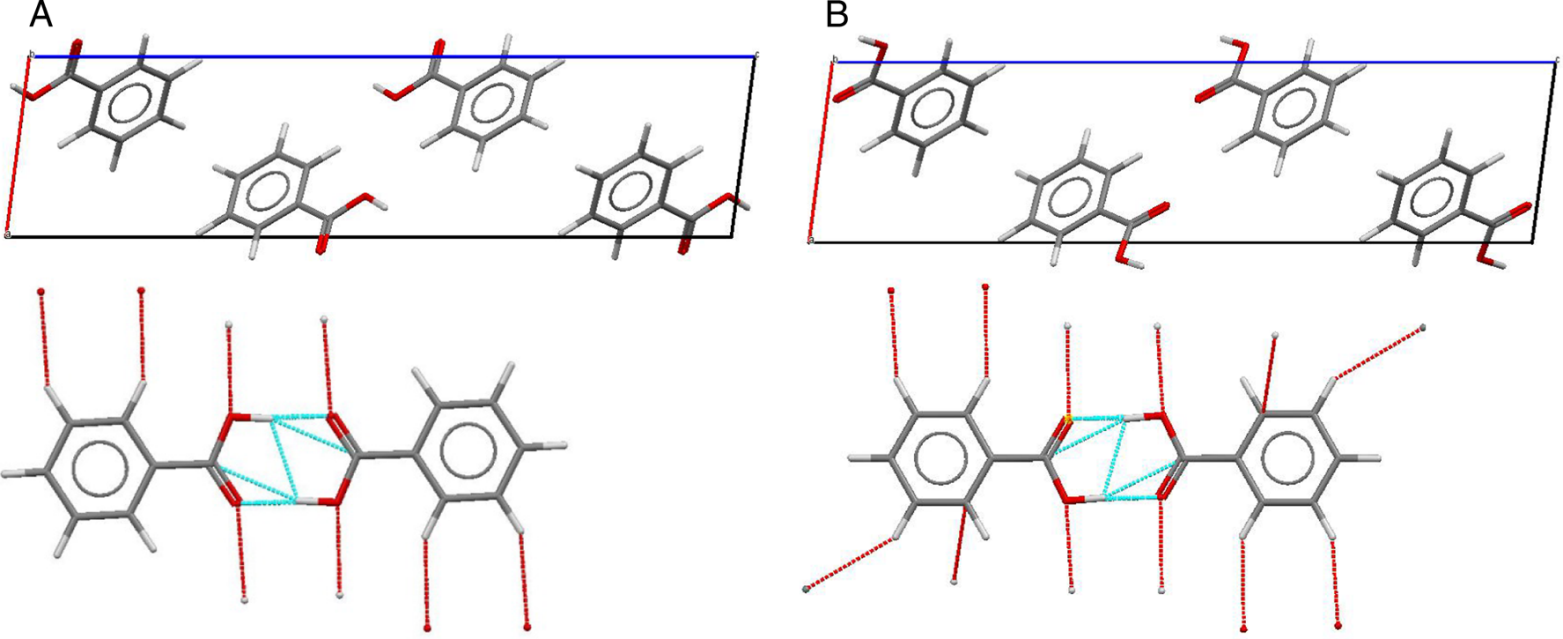
Representation of two isomeric forms of benzoic acid crystals (view along b direction). Close contacts and hydrogen bonds are marked by red and blue lines, respectively

Despite the fact that all structures deposited in CSD underwent refinements according to protocols related to X-ray diffractions data processing, all crystal–structure models considered here were re-optimized within Dmol^3^ [24] module implemented in Accelrys Material Studio 7.0 package [25]. This step is important for several reasons. First of all refinements details are not the same for structures deposited by different authors and some are incomplete or flawed. For example, the inspection of thermal extension of cell volume suggests that file BENZAC12 is probably erroneous either in reported temperature (123 K) or measured volume. If temperature is provided accurately the observed linear trend suggests that more probably cell volume is 10.2 Å^3^ higher. Furthermore, the existence of two possible isomeric forms related to tautomerisation within *C*_2_^2^(8) synthon requires precise control of carboxylic-group conformations. Indeed, as it was documented in Fig. 1 there were formallytwo possible types of crystals which differed by hydrogen atom positions. These two types of arrangements, although fulfilling general definition of H-tautomers do not lead to polymorphism of benzoic acid. Rather, these structures corresponding to disordered hydrogen atoms over two sites with varying occupancies exhibit inherent heterogeneity of solid state. Here, these two contributions are analyzed in detail. Thus, based on data provided in CIF two alternative forms were generated for each CSD deposited at all eight reported pressures in the range of 0.10–2.21 GPa. Then the intra-molecular geometric parameters of benzoic acid of 15 structures were pre-optimized. The same level of computations was applied for all systems, namely PBE [26] density functional approach with DNP version 3.5 basis set [27]. This double numerical basis set incudes polarization d-function on all non-hydrogen atoms and additionally p-function on all hydrogen atoms. Such representation is essential for proper hydrogen bonding computations. The Grimme [28] corrections for dispersion contribution were used. The fine option was used for integration accuracy and SCF tolerance (<10^−6^). All electrons were included in the core treatment. The fine option for orbital cutoff quality was set. The cell parameters were unchanged and kept constant as provided by experiments. The structures obtained in this step were deposited in Supporting materials (see Table S1).

### Structural characteristics

As a measure of structural changes imposed by pressure the extent of π-electrons delocalization was used. Among many alternative definitions of the aromaticity the geometric indices were selected [29–32]. According to the original definition [30, 31] the π-electrons resonance occurring within an aromatic ring can be quantified as a weighted sum of squared differences between actual lengths of bond forming the ring and reference values for model aromatic molecule,

1 $$ \mathrm{HOMA}=1\hbox{-} \frac{1}{\mathrm{n}}{\displaystyle \sum_{\mathrm{i}}\upalpha {\left({\mathrm{R}}_{\mathrm{opt}}\hbox{-} {\mathrm{R}}_{\mathrm{i}}\right)}^2=1\hbox{-} \mathrm{E}\mathrm{N}\hbox{-} \mathrm{G}\mathrm{E}\mathrm{O},} $$

where n is the number of CC bonds and α = 257.7 stand for the empirical normalization constant. This value is chosen to give HOMA = 0 for a model nonaromatic system and HOMA = 1 for a system where all bonds are equal to reference ones, R_opt_ = 1.388 Å [31]. According to Krygowski et al. [32] it is possible to define two independent contributions to HOMA index. The one denoted as GEO represents a decrease of aromaticity due to an increase of the bond length alternation within the ring. The second one, EN, is related to the size of the ring and reflects an increase of the average bond length in a given structure. Since the only data indispensable for such geometric characteristics require the Cartesian coordinates the computations are straightforward for both experimental and optimized geometries.

### Crystal energy computations

The optimized structures were used for thermodynamics computations on the same level of theory. The total energy of the system (E_cryst_) treated with periodic DFT approach can be used for the lattice energy estimation defined as the difference between energies of a monomer in crystal (i.e., crystal energy per unit cell E_cryst_/Z) and in the gas phase (E_mon_):

2 $$ {E}_{latt}=\frac{E_{cryst}}{Z}-{E}_{mon}, $$

where Z is the number of molecules in one unit cell and in the case of benzoic acid crystals Z = 4. Since the vibrational contributions have non-negligible effect on the lattice energy the periodic DFT calculations were used for computing unit cell normal modes of vibration. The same method was used as for optimization. The obtained data can be used for computations of the following relative values.

3 $$ \varDelta ZP{E}_{latt}=\frac{ZP{E}_{cryst}}{Z}-ZP{E}_{mon}\;{E}_{vib, latt}=\frac{E_{vib, cryst}}{Z}-{E}_{vib, mon} $$

The symbol ΔZPE_latt_ represents the change of the zero point vibrational energy of the monomer in the lattice with respect to the gas phase. Similar change characterizing thermal excitation of the molecular and lattice vibrations is denoted as ∆E_vib_.

Additionally the pair-wise additive model was assumed for crystal energy computation. Although, this seems to be a crude approximation it is surprisingly effective and accurate if compared to experimental sublimation enthalpies. The lattice energy of a crystal is simplified by the sum of dimers stabilization energies. Taking advantage of the Mercury software [33] being a part of CSD package [3], the closest proximity of benzoic acid molecule in crystals was considered. The nearest neighborhood was defined by separation distance between two monomers not exceeding the sum of van der Waals radius augmented by 1 Å of any pair of atoms belonging to either of the monomers. The default values of van der Waals radii used in Mercury were applied [33]. This is accepted as the standard procedure [33] defining the molecular shell (MS) within crystal. In the simplest case with just one compound per asymmetric unit (Z’ = 1) all molecules in the crystal are supposed to be structurally and energetically identical as is the case for benzoic acid solids. In order to reduce the overall cost of computation the number of unique arrangements of two benzoic acid molecules were identified. For this purpose each dimer found within MS was classified according to symmetry operators defining monomers position in the crystal. Since each allowable setting of a space group is characterized by a set of symmetry operators (SO) indicating the relative positions of symmetry-equivalent atoms in the structure, also pairs conformations can be expressed in terms of relative transformation vectors (RTV). Each RTV is simply the difference between SO of monomers and in the case of molecular shell the number of RTV is simply equal to number of neighbors (NN). Molecular shell very often possesses redundant information since several dimers can be characterized by the same intermolecular interaction energy (IIE). Thus, unique pairs (N_up_) are defined by both unique RTV and IIE. This reduces the number of possible pairs necessary for constituting molecular shell, super cell or bulk crystals since only distinct symmetry allowed patters and energetically distinct pairs are considered. The N_up_ value has the simple meaning of number of pair interactions indispensable for additive construction of the whole crystal stabilization energies. Thus, molecular shell defines all possible pairs occurring in the crystal and lattice energy is then simply the sum of unique pair interaction energies (ε_IIE_) weighted by their occurrence (n_ij_), namely E_latt_ ≈ E_MS_ = 0.5⋅Σn_ij_⋅ε_IIE_ (ij) (factor 0.5 is used for avoiding double counting of intermolecular interactions in crystal).

### Intermolecular interaction energy and its decomposition

Intermolecular interaction energies of unique pairs formed by benzoic acid were estimated using first principle approach. Particularly two functionals were used, wB97XD [28, 34] and M06x [35], along with aug-pvDZ basis set (aDZ) and ET-pVQZ (even tempered valence quadruple zeta with polarization functions), respectively. For this purpose G09 [36] and ADF packages [37] were utilized. Additionally, the Kohn-Sham DFT approach was used for assessments of detailed energetic contributions to ε_IIE_ values. The energy decomposition analysis (EDA) [38] offers simple and straightforward way of decomposing intermolecular interactions into meaningful contributions:

4 $$ \varDelta {E}_{IIE}=\varDelta {E}_{TPR}+\varDelta {V}_{EL}+{E}_{OI}=\varDelta {E}_{TSI}+{E}_{OI}. $$

The total Pauli repulsion ΔE_TPR_ comprises the destabilizing interactions between occupied orbitals and is responsible for any steric repulsion. The classical electrostatic interactions between the unperturbed charge distributions of interacting fragments is denoted as ΔV_EL_. The orbital interaction ΔE_OI_ accounts for charge and polarization due to bonding. The sum of Pauli repulsion and electrostatic attraction is often termed the total steric interactions E_TSI_. The EDA scheme defined in Eq.4 was successfully applied for analysis of a variety of chemical systems [39, 40]. Thus, the direct advantage of using additive model is the possibility of application of EDA for studying the origin of lattice stabilization.

## Results and discussion

The characteristics of energetic patterns of benzoic acid crystals was done in three steps. Initially, all available crystal structures deposited in CSD were pre-optimized in two alternative conformations. Obtained crystals were used for molecular shell preparation. Then unique contacts within molecular shell (MS) were identified and intermolecular interaction energies (IIE) were computed based on first principle approach. Besides, decomposition of IIE into contributions derived through energy decomposition analysis (EDA) along with selected structural and electronic descriptors allowed to quantify pressure related alterations of benzoic acid crystal properties.

### Crystal structures of benzoic acid in CSD

There are 20 CIFs of benzoic acid crystals deposited in the CSD. BA does not exhibit polymorphism and all structures correspond to monoclinic crystals system characterized by P21/c space group. There are three deposits in CSD of benzoic acid crystals measured at ambient conditions, namely BENZAC, BEZAC01, and BENZAC02 [17, 18, 41]. It is worth mentioning that there are some discrepancies among crystals details. For example the cell volume reported for these three structures is equal to 616.733 Å^3^, 619.15 Å^3^, and 613.955 Å^3^, respectively. Furthermore, not only variation of cell parameters is to be noticed but also significant changes of all crucial structural features affecting intermolecular interactions are to be mentioned. For example the main *C*_2_^2^(8) synthon geometry is significantly different in these structures since hydrogen bond length is equal to 2.616 Å, 2.633 Å, and 2.627 Å for these structures. This is the reason why the geometry optimization is required instead of direct use of CIF content. Optimization of benzoic acid molecular structures without changing cell parameters leads to a much better congruency of molecular geometry (2.601 Å, 2.608 Å, and 2.603 Å, respectively). Additionally, the hydrogen atom position within *C*_2_^2^(8) synthon of benzoic acid and its derivatives is questionable [42] due to concurrency between electronegative centers leading to hydrogen atoms disorder [17, 18, 41–44]. Often two alternative coordinates of hydrogen atoms are provided in CIF files for two possible tautomeric forms. As was documented experimentally using single crystal neutron diffraction [42] the crystal structures of benzoic acid as other carboxylic acids comprise dimers bound via two pairs of hydrogen bonds. Thus, formally two distinct types of crystals are expected comprising two types of configurations as documented in Fig. 1. These two forms have different relative site occupancies affected by temperature with form A being promoted in cooler conditions. It is important to note the XRD spectra of all these crystals are nearly indistinguishable. Interestingly, there are non-negligible differences in the molecular shell of these two isomeric crystals. Thus, pressure related changes of benzoic acid crystals were described for both tautomers. The CSD reports also measurements of benzoic acid crystals at non ambient conditions. There are seven deposits at higher pressures up to 2.21 GPa and nine structures measured after cooling down to 5 K. Here the former set is used for exploring the pressure related changes of structural energetic and electronic properties of benzoic acid crystals.

### Accuracy of IIE predictions

**Fig. 2:**
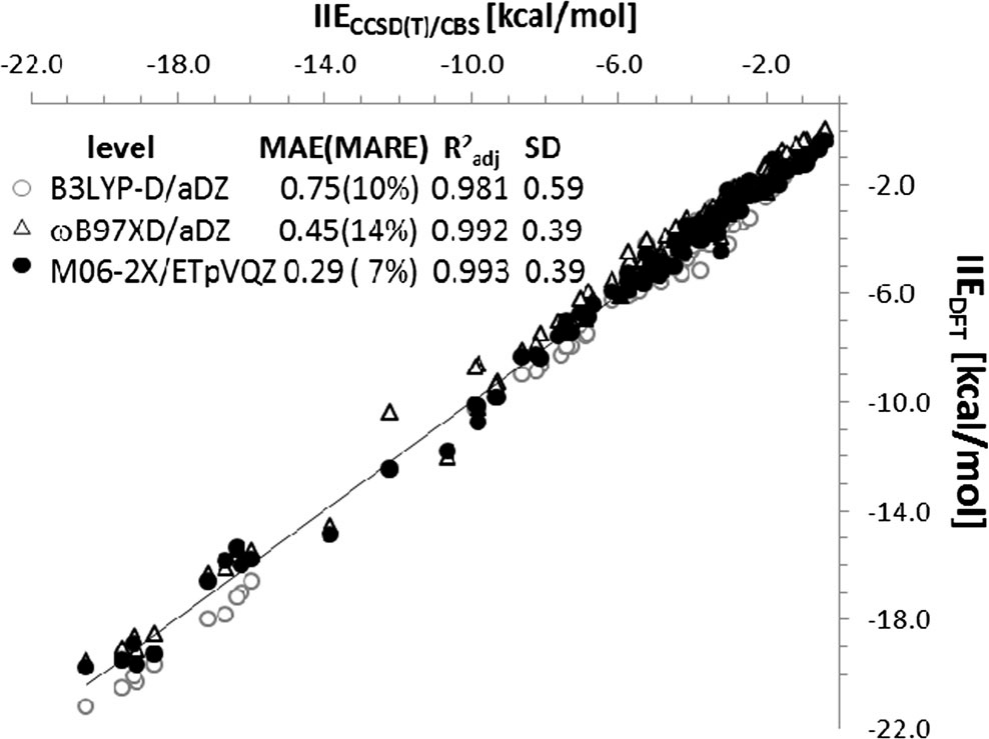
The correlation of IIE energies estimated for the set comprising S66, S26, and X40 Hobza benchmarks [49, 50]. Mean average error (MAE), mean average relative error (MARE), square of adjusted correlation coefficients and standard deviations are provided along with regression parameters

Before detailed characteristics of intermolecular interactions in crystals leading to lattice energy prediction the level of computations is to be selected. As was mentioned in the methodology section the pair additive model is used in this study. This seems to be a crude approximation of crystal energetics, but the pairwise addictive model for crystals energies characteristics was successfully used in a variety of contexts. For example polymorph stability was analyzed throughout crystal energy landscapes concept [44] in which intermolecular interactions stand for driving forces of organic molecule arrangements. The recognition of basic structural motifs was introduced in the case of fused hydrocarbons crystal packing by strongly bonded fragments of crystals [45]. Also, additive model can be used to assess the lattice energy of a series of experimental and model benzoic acid/benzamide cocrystals [46]. Nowadays, the additive model was used for explanation of the origin of orientation effect observed on polar surfaces after droplet evaporation crystallization [47, 48]. These encouraging attempts of simplification of cooperatives in crystals were motives for studying of consequence of external stress on crystals structure of benzoic acid in terms of pair interactions. However, the quality of IIE strongly depends on accuracy of method applied for super-molecule computations. There are many available DFT functionals, which can be successfully used for pair interaction predictions. Here, two methods were selected as available in G09 [36] and ADF [37] packages. The training set was quite extensive and comprised 132 pairs formed by conjunction of S66, S26, and X40 sets of Hobza benchmarks for the gas-phase intermolecular interactions [49, 50]. This exhaustive set of molecular pairs of great diversity covers a broad range of stabilization energies and also types of interactions. The results of performed tests are presented in Fig. 2.

As one can see all applied methods perform quite well and met the so-called chemical accuracy demand what is suggested by provided values of adjusted correlation coefficients, mean average deviation error (MAE), and mean average relative error (MARE). It seems that M06-2X functional implemented in ADF package augmented with quite extended basis set ET-pVQZ is the best suited for IIE estimation among tested approaches. The good description of the intermolecular interactions in such variety of pairs as found in the testing set is promising prognostics for application to crystals energetics computations. Thus, M06-2X/ET-pVQZ level is used across this study as sufficiently accurate for description of pair IIE. For computations of lattice energy of benzoic acid crystals the pairs of molecules were generated based on molecular shell formed by 16 molecules. However, among 15 intermolecular contacts only nine are distinct and univocally define all energetic patterns within both bulk crystal at either pressure studied here. The values of intermolecular interactions of these contacts are provided in Supporting materials in Table S2. For validation of reliability of additive model the experimental values of sublimation enthalpies were used. Since the sublimation enthalpy, ΔH_sub_ (T), of a crystal is a direct measure of the lattice energy these data are very often used for theoretical models verification [51] by the following simplified formula:

5 $$ \varDelta {H}_{sub}(T)=-{E}_{latt}-2RT, $$

where T is the temperature at which the sublimation enthalpy is measured, and R is the gas constant. It is worth mentioning that there are systematic uncertainties associated with the measurement of sublimation enthalpies of solids and it is common to notice quite large variances in sublimation enthalpy of the same compound [52]. Benzoic acid is used as internal standard thermo gravimetric measurements and for this purpose precise heats of phase changes are indispensable. This is the reason why measurements were repeated by different authors using a variety of experimental approaches. There were published 47 measurements of benzoic acid sublimation enthalpies [52]. After adjustments to standard temperature the average value is equal to 21.4 kcal mol^-1^ with averaged standard deviation 2.3 kcal mol^-1^. The corresponding value of lattice energy equals −22.5 ± 2.3 kcal mol^-1^. It is worth emphasizing that additive model used for lattice energy prediction is very effective in the case of benzoic acid crystals. The perfect matching between computed and experimental values of lattice energy is a fortunate circumstance allowing for application of additive model and associated EDA for studying of pressure related changes benzoic acid crystals.

### Pressure-imposed structural alterations

**Fig. 3:**
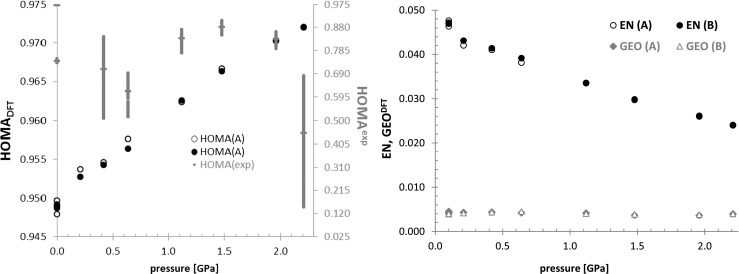
The variation of geometric aromaticity measures characterizing benzoic acid as a function of pressure imposed on benzoic acid crystals

There are many ways of expressing structure changes of crystals under non-ambient conditions. The first and direct consequence of pressure are unit-cell parameters. The inspection of experimental values above 0.1GPa suggest a fairly linear trend in reducing of the size of the unit cell and rise of β angle. Besides, the pressure must also lead to alterations of both molecular geometry and packing motives. Indeed, as one could expect the *C*_2_^2^(8) synthon characterized by two very strong hydrogen bonds is sensitive to the external stress. The observed systematic shortening of hydrogen bond lengths was confirmed by corresponding band shifts on Raman spectra [21]. This is quite expected since the main source of density increase of compressed crystals is the void shrinking by imposing closer proximity between monomers. Due to the fact that for benzoic acid there is not observed any transition state in studied range of pressure, the closer packing of the molecules is the major consequence of the external stress. However, instead of detailed analysis of bond length and angles changes associated with higher packing at elevated pressure the structure alterations are expressed here in terms of π-electron delocalization. The applicability of this approach for monitoring of inner geometry change of aromatic systems is due to the fact that the manifestation of π-delocalization is associated by reduction of bond-length alternation, as compared to their acyclic unsaturated analogues. Since benzoic acid is supposed to be a rather strong aromatic compound [29] the application of HOMA as ultra-sensitive to even minute geometry changes seems to be both informative and straightforward. The effectiveness of harmonic oscillator model of aromaticity, HOMA [24] was already documented as a very useful and direct method for inspection of accuracy of experimental structures deposited in crystallographic databases [53]. It was demonstrated that even extra fine resolution (≤1.0 Å) of X-ray diffraction measurements of macromolecular crystals is not sufficient for direct use since estimation HOMA values based on Cartesian coordinates provided by PDB files of protein and DNA crystals lead to unrealistic values. This imposed serious limitations on the direct use of Cartesian coordinates and even the best available PDB files cannot provide structures of acceptable accuracy. Here HOMA values were computed for both optimized geometries and ones provided by deposited CIF. Since fractional coordinates are often provided with values of standard deviations (SD) quantifying limited precision of X-ray measurements the sensitivity of HOMA values to such uncertainties were quantified by computing corresponding distributions of this geometric index. For this purpose all possible variations of fractional coordinates according to (+/−) SD were computed and after conversion to Cartesian coordinates were used for HOMA values estimation. The obtained spans of geometric index of aromaticity were included in Fig. 3.

**Fig. 4:**
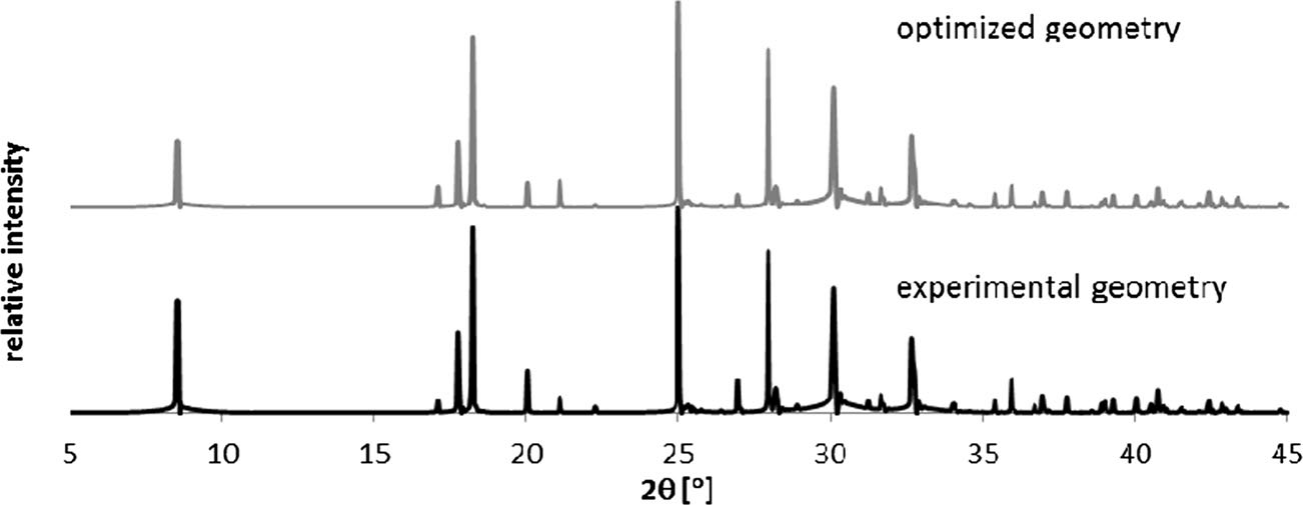
The X-ray spectra generated based for BENZAC19 using directly CIF content and after geometry optimization using periodic DFT

First of all, it is visible that geometry of benzoic acid ring is sensitive to external stress which is associated with increase of HOMA values. Strong aromaticity of benzoic acid is even enhanced by elevation of pressure for both considered crystal conformers if DFT-derived geometries are used. The hydrostatic pressure affecting crystal density leads to increase of π-electron delocalization. However, the application of geometries directly provided in the CIF leads to quite a different picture. First of all the absolute values of HOMA are much lower suggesting dramatically lower aromaticities. Even at ambient conditions there is discrepancy between obtained HOMA values associated with different refinement protocols applied by different authors. The structure deposited as BENZAC13, which was measured at 0.21 GPa is characterized by as low HOMA values as 0.617. Such a small value is typically addressed to very low aromatic character. Although there is observed systematic and parallel rise of aromaticity predicted both by experimental and optimized geometries of BA the more realistic and consistent are the latter values. Furthermore, the BENZAC15 structure is most probably wrong since obtained HOMA value has unphysical value (−1.414). Also distribution of this geometric index is out of the expected range and most probably is not related merely to low precision of X-ray measurements. Thus, it was not included and that is why it was not included in Fig. 3. On the other hand the qualitative agreement in trends of HOMA is broken around 2 GPa. The nearly linear increase of HOMA^DFT^ values is in sharp contrast with the trend of HOMA^exp^. This raises the question if refinement protocols applied during X-ray data processing should be further refined by periodic DFT computations. It is interesting to note that despite of discrepancies in geometric details the X-ray diffraction spectra obtained based on original and optimized crystals are indistinguishable. Indeed in Fig. 4 there are presented spectra computed with an aid of Reflex module of MS7.0 using experimental data and ones coming from optimization procedure. Since these plots are essentially identical the X-ray experiments should not be the only source of structural information. This is why many high-pressure crystallographic experiments are augmented by spectroscopic measurements [20, 21]. Furthermore, the limited precision of X-ray experiments lead to a broad range of HOMA values. This index is extremely sensitive to even small geometry variations of the ring and seems to be suited for precise ring geometry validation, as was mentioned beforehand [53]. On the other hand the observed discrepancies both in the HOMA values and pressure related trends cannot be addressed merely to X-ray experiments precision.

On the right panel of Fig. 3 there are also presented plots characterizing two contributions to HOMA. The most dominant effect on HOMA comes from EN component. The small and constant values of GEO suggest that pressure influences are of isotropic character and do not lead to bending, torqueing, and stretching of the ring. This is not so obvious since shortening and enhancement of hydrogen bonding might result in elongation of the ring length. Apparently, this is not the case for the benzoic acid molecule at studied range of pressures. The increase of pressure leading to lowering of EN contribution is associated with reduction of BA ring diameter. Indeed, decrease of the total area of the ring is observed along with reduction of the sum of ring bond lengths. Thus, aromatic ring present in the benzoic acid crystals is strongly affected by pressure, which imposes shrinking of the ring but not its regularity of the shape.

### Pressure related energy changes

**Fig. 5:**
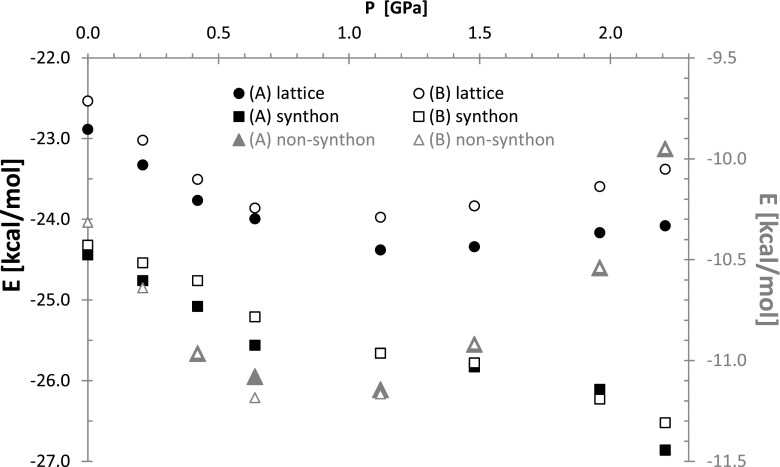
Pressure induced lattice energy change of benzoic acid crystals in two tautomeric forms computed using additive model on M06-2X/ET-pVQZ level. The contributions coming from synthon (left abscissa) and the rest of the pair interactions (right abscissa in gray) are provided

The pressure-dependent alterations of benzoic acid crystal packing and molecular structure must be associated with corresponding energetics of the lattice. Although one can anticipate that rising pressure leads to destabilization of crystal, the observed trend in case of benzoic acid is non-monotonous. Indeed, as is presented in Fig. 5 initial increase of hydrostatic pressure reduces lattice energy suggesting increase of its stability. If pressure exceeds 1 GPa the trend is reversed and non-negligible destabilization contributions are observed. However, even at the highest pressure investigated (2.21 GPa) the lattice energy is lower compared to values characterizing benzoic acid crystals at ambient conditions. Second conclusion derived from data provided in Fig. 5 is related to synthon contribution to crystal stabilization. Interestingly, in the whole range of applied pressures not only the major contribution to the stabilization of crystals comes from interactions within main synthon but this attraction force is strengthened with increases of pressure. Even at the highest pressure for which crystal data are available the interactions between two carboxylic groups are stronger than at ambient conditions. This is a rather unexpected observation suggesting that geometry and arrangements of benzoic acid molecules in crystals at normal pressure is not energetically the most efficient and pressure shifts geometries of benzoic acid molecules toward more energetically favorable conformation. The reason why such energetically more advantageous BA arrangements are not adopted at normal pressure must then come from the rest of interactions, which are responsible for the observed non-monotonous change of lattice energy with the rise of pressures. Indeed, in Fig. 5 the right ordinate axis quantifies the sum of all non-synthon interactions and plots marked by gray triangles represent the trend of such lattice energy response to the external stress. Thus, although each individual pair IIE of non-synthon type are much smaller in comparison with synthon interactions (see Table S2 in Supporting materials) their sum stands for cumulative network response to elevation of pressure. This response is still more attractive if compared to ambient pressure but is systematical reduced with the rise of pressure. It is also interesting to note that both studied isomers are not energetically identical and also their sensitivity to external stress is not the same. Presented in Fig. 5 plots of lattice energies of both isomers suggests that the more stable conformer (A) is slightly less prone to changes imposed by the rise of pressure and this difference is even enhanced in more extreme conditions.

**Fig. 6:**
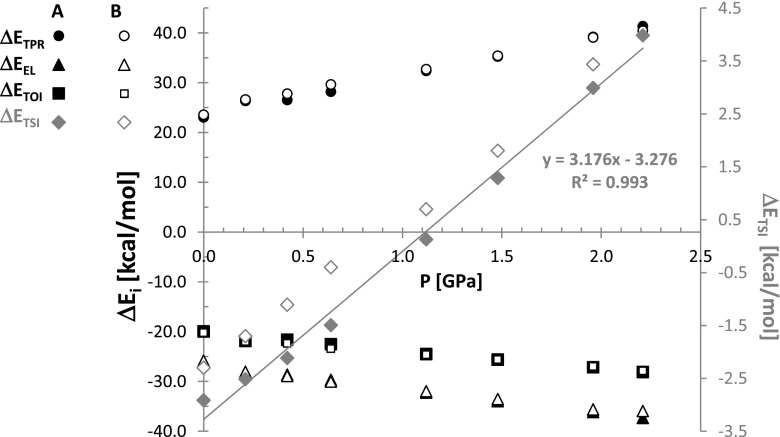
Pressure related changes of energy components of benzoic acid crystal lattice

**Fig. 7:**
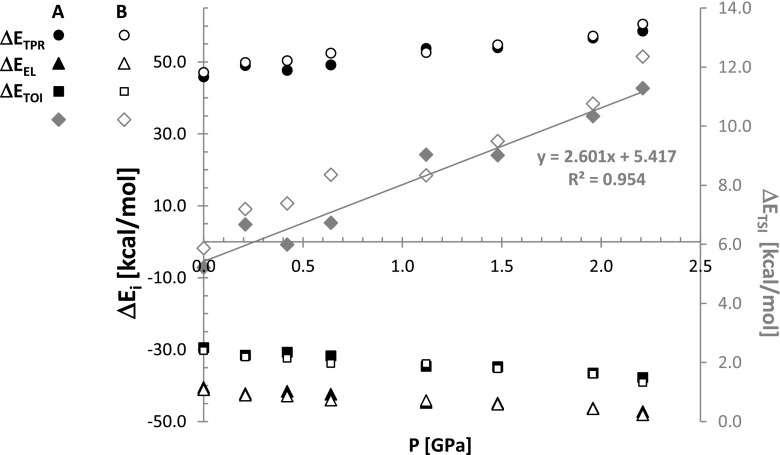
Pressure related changes of energy components of synthon found in benzoic acid crystals

It is interesting to see what is the origin of the trend change near 1GPa. For this purpose the decomposition of energy contributions into components was done according to Morokuma-Ziegler bond energy decomposition scheme [38]. In Figs. 6 and 7 there are presented results of such an analysis for the lattice energy and additionally for *C*_2_^2^(8) synthon alone.

As one can directly infer from Fig. 6 there is monotonous change of all considered contributions with the rise of pressure. Indeed, significant increase of destabilization contribution of total Pauli repulsions is associated with systematic decrease of both electrostatic term as well as orbital interaction energies. It is worth mentioning that the former contribution is stronger than the latter in the whole range of studied pressures. Interestingly, the total steric interaction term accounting for the sum of Pauli and electrostatic contributions, changes its sign slightly above 1 GPa. Since this value is smaller than total orbital interaction energies the overall stabilization of crystal lattice is still high. However, from this pressure threshold the total steric interactions become positive. The observed trend of ΔE_TSI_ is almost a linear function of pressure with correlation coefficient as high as 0.996. Thus, benzoic acid crystals raises its stabilization with an increase of pressure due to overwhelming contribution coming from the orbital interactions despite destabilization introduced at high pressure by steric repulsions that are mainly of Pauli nature. In Fig. 7 the results of energy decomposition of *C*_2_^2^(8) synthon interactions is presented. It is evident that although monotonous change of all contributions is visible, the destabilization coming from steric interactions arises even if slight stress is introduced. This suggests that the observed rise of synthon stabilization with increase of pressure is due to orbital interactions, despite steric hindrances. All above conclusions about the role of particular contributions to lattice energy holds for both considered conformers.

**Fig. 8:**
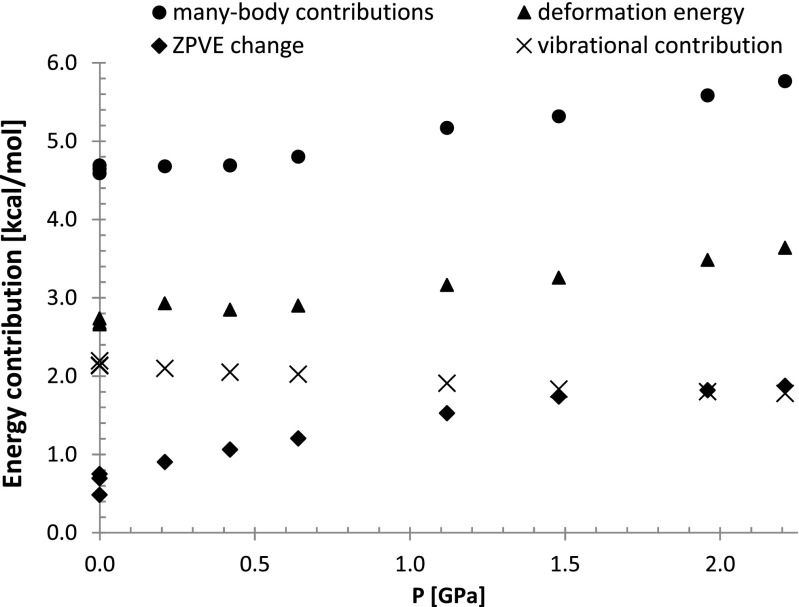
Pressure imposed alteration of relative ZPVE, monomer deformation, and non-additive many-body interactions within the benzoic acid crystals. All relative values are estimated with respect of isolated monomer in the gas phase at the same level of theory using PBE functional

Besides two-body contributions it is interesting to see how sensitive to pressure is the change of many-body interactions. They can be estimated by adopting the approach suggested by Tsuzuki et al. [54]. Since the total energy of the crystal accounts for both additive and non-additive terms the subtraction from the former the sum of pairs interactions provides quantification of the many-body interactions. Of course the same level of theory is used in both cases. This is why pair interactions within molecular shell were recalculated using the same functional as used for the periodic DFT calculations. The obtained results are presented in Fig. 8, which also comprise plots characterizing zero point energy change, vibrational energy contribution, and the deformation energy of a molecule associated with the crystal formation. The latter value corresponds to the change of energy of free monomer after geometry alterations imposed by crystal environment. As one can infer from Fig. 8 all contributions are positive and show similar variations with pressure change except from the thermal vibration term. The latter is slightly decreased with the elevation of external pressure. The main conclusion derived from Fig. 8 is that in the range of studied pressure all these contributions are rather insignificant since they do not differ by more than 1 kcal mol^-1^ if compared to ambient conditions. This is a fortunate circumstance making computations of crystal properties at elevated pressure much less demanding and applicability of additive model more reliable.

## Conclusions

The hydrostatic pressure imposes many changes to benzoic acid crystals. Since carboxylic acids exhibit inherent disorder in protons occupations within *C*_2_^2^(8) synthon [55] both possible conformations were considered explicitly. The directly observed variations of cell parameters and consequently cell volume are associated with many other changes including energetic, geometric, and electronic features. Some of these alterations are not directly predictable and computations lead to non-trivial conclusions. First of all non-monotonous change of lattice energy are noticed with the rise of pressure. Namely, the increase of stabilization up to 1GPa is followed by systematic decrease of lattice energies after extending the hydrostatic pressure. Also the observed increase of *C*_2_^2^(8) synthon stabilization interaction with increase of pressure is somewhat surprising suggesting that the monomers conformation in crystals at ambient conditions is far from global minimum. Thus, non-negligible and non-linear variation of the cooperativeness in the lattice is a crucial factor contributing to overall stability. Besides, energy decomposition analysis revealed the origin of the observed energetics trends associated with pressure change. This is the contribution coming from total steric interactions that determine the overall trend of lattice energy change with rise of pressure. Interestingly, orbital interactions systematically increase with rise of pressure in the whole range of studied conditions. Although this is also associated with increase of Pauli repulsions the relative values of these two contribution changes the sign around 1 GPa. The energy consequences of increasing pressure obviously originate from structural changes. Among many parallel changes the π-electron delocalization expressed in terms of geometric measures exhibits an interesting behavior. The high aromaticity of benzoic acid is significantly reduced at higher pressures. However, the stress imposed on BA crystals leads to symmetric aromatic ring shrinking without affecting the alteration of bod lengths. Although crystals can be anisotropic in many respects in the case of benzoic acid intermolecular interactions affected by pressure lead to quite isotropic field. Besides, many other changes are observed within BA crystals with increase of pressure. Among others it is interesting to note the sizable reduction of hydrogen bond length within *C*_2_^2^(8) synthon, rise of OH length in carboxylic group, increase of dihedral angle defining geometry within carboxylic group, noticeable and linear increase of Laplacian and electron densities estimated for ring critical points of both aromatic ring and synthon and also increase of frontier orbital energies leading to a rise of hardness and chemical potential. Since benzoic acid is a representative of a very important class of crystals one can expect that discussed observation of stress consequences imposed on crystals can also be extended to other crystals of similar type. Unfortunately, benzoic acid is the only aromatic acid for which there are available detailed information about crystal parameters at high pressures and perhaps not all conclusions can be generalized and extended for other systems with different symmetry groups. Furthermore, some discrepancies were noticed between experimental and computed properties. Strong disagreement between HOMA^DFT^ and HOMA^exp^ values, although quite understandable at lower pressures, is surprisingly high at more extreme conditions suggesting some limitations of periodic DFT computations. The experimentally observed braking of molecules at very high pressures will probably not be accounted in this approach. This suggests limiting of periodic DFT computations implemented in DMol3 to modest pressures. Despite these limitations the pressure consequences on crystals properties seems to be interesting and worth further explorations.

## Electronic supplementary material

Below is the link to the electronic supplementary material.

ESM 1 (DOC 41 kb)

ESM 2 (DOC 38 kb)
