# Supplementary material for: Pressure-imposed changes of benzoic acid crystals
Source: J Mol Model. 2015 Mar 13;21(4):83. doi: 10.1007/s00894-015-2635-z (PMC4357648; doi:10.1007/s00894-015-2635-z)
Supplement: Supplementary file 1 — (DOC 41 kb) [file 894_2015_2635_MOESM1_ESM.doc]

**Table S2** Energetically distinct contacts between benzoic acid molecules defining all possible pairs in crystals. All energies expressed in kcal/mol correspond to M06-2X/ET-pVQZ computations. Provided uncertainties are estimated based on three available crystals measured at ambient conditions (BENZAC, BENZAC01, BENZAC02). The value of estimated Lattice energy is provide in bold style.

|  | **pair** | **n_i_** | **A** | **B** |
| --- | --- | --- | --- | --- |
| 1 | -x,-y,-z | 1 | -24.4±0.1 | -24.5±0.1 |
| 2 | x,1+y,z | 2 | -3.1±0.6 | -2.7±0.1 |
| 3 | 1-x,1-y,-z | 1 | -2.2±1.8 | -0.5±0.0 |
| 4 | -x,1-y,-z | 1 | -3.2±0.4 | -3.9±0.1 |
| 5 | -1+x,y,z | 2 | -1.9±0.6 | -1.7±0.0 |
| 6 | 1-x,1/2+y,1/2-z | 2 | -0.9±0.4 | -0.9±0.0 |
| 7 | -x,-1/2+y,1/2-z | 2 | -0.6±0.3 | -0.7±0.0 |
| 8 | 1+x,1+y,z | 2 | -0.8±0.5 | -1.7±0.0 |
| 9 | 1-x,-y,-z | 1 | 0.4±0.7 | -0.7±0.0 |
|  | **E_latt_** |  | **-22.9±0.1** | **-22.6±0.1** |
